# Network Analysis Reveals the Recognition Mechanism for Dimer Formation of Bulb-type Lectins

**DOI:** 10.1038/s41598-017-03003-5

**Published:** 2017-06-06

**Authors:** Yunjie Zhao, Yiren Jian, Zhichao Liu, Hang Liu, Qin Liu, Chanyou Chen, Zhangyong Li, Lu Wang, H. Howie Huang, Chen Zeng

**Affiliations:** 1 Institute of Biophysics and Department of Physics, Central China Normal University, Wuhan, 430079 China; 2 Research Center of Biomedical Engineering, Chongqing University of Posts and Telecommunications, Chongqing, 400065 China; 3 Department of Physics, The George Washington University, Washington, DC 20052 USA; 4 Department of Electrical and Computer Engineering, The George Washington University, Washington, DC 20052 USA; 5 School of Life Sciences, Jianghan University, Wuhan, 430056 China

## Abstract

The bulb-type lectins are proteins consist of three sequential beta-sheet subdomains that bind to specific carbohydrates to perform certain biological functions. The active states of most bulb-type lectins are dimeric and it is thus important to elucidate the short- and long-range recognition mechanism for this dimer formation. To do so, we perform comparative sequence analysis for the single- and double-domain bulb-type lectins abundant in plant genomes. In contrast to the dimer complex of two single-domain lectins formed via protein-protein interactions, the double-domain lectin fuses two single-domain proteins into one protein with a short linker and requires only short-range interactions because its two single domains are always in close proximity. Sequence analysis demonstrates that the highly variable but coevolving polar residues at the interface of dimeric bulb-type lectins are largely absent in the double-domain bulb-type lectins. Moreover, network analysis on bulb-type lectin proteins show that these same polar residues have high closeness scores and thus serve as hubs with strong connections to all other residues. Taken together, we propose a potential mechanism for this lectin complex formation where coevolving polar residues of high closeness are responsible for long-range recognition.

## Introduction

Plant lectins are carbohydrate-binding proteins that are abundant in seeds, flowers, leaves, roots, and other vegetative non-storage tissues. These lectins are recognized as plant defense proteins because they can specifically target the surface glycan of the epithelial cells lining the intestinal tract of insects and some herbivores^1–5^. The harmful and toxic effects of glycan binding vary from slight discomfort to even death. As our understanding of lectin-carbohydrate interaction grows, the biological applications of lectins also become much more diverse^6^. Besides the anti-insect activity, the plant lectins were used as molecular tools to study host-pathogen interactions, cell development and signaling, and many others in biomedical applications^7–15^.

Plant lectins have been classified into 12 families based on their sequences, fold structures, and carbohydrate binding motifs^16–19^. The most general features of plant lectins are as follows. (1) The carbohydrate binding domains are evolutionarily related; and (2) lectins typically form dimer or oligomer for their biological activities. However, the recognition mechanism for lectin dimer or oligomer formation remains poorly understood and is the subject of our study.

Such study is becoming feasible given the rich data sources on plant lectin families. First, the known structures of most lectin complex deposited in Protein Data Bank (PDB) enable molecular dynamic simulations and the associated correlation network analysis^16, 17^. Graph theory concepts such as betweenness and closeness can be brought to bear in identifying critical residues for complex formation. Second, there happen to be abundant single-domain and double-domain lectins in plant genomes for us to distinguish these critical residues in terms of whether they are for short-range or long-range recognitions. In the crowded environment of a cell, one single-domain lectin may need to find the other single-domain interacting partner at a distance to form a dimer. However, such a long-range recognition is no longer required in double-domain lectin where two single-domain lectins are fused into one protein with a short linker and are always in close proximity. We thus perform statistical inference in terms of direct coupling analysis (DCA) on sequence evolution for single- and double-domain lectins to probe the conservation as well as coevolution of the putative critical residue pairs from multiple sequence alignments. These methods together allow us to uncover the protein-protein recognition mechanism.

In this article, we select the bulb-type lectins for detailed computational analysis to gain insights into lectin recognition mechanism^18–28^. The bulb-type mannose-binding lectin is a beta-prism type II structure. The single-domain or monomer protein contains antiparallel beta-strands with 3-fold symmetry. Two monomers can assemble into a dimer structure by inserting their C-terminal beta-strand tails into each other to form beta-sheets. This particular lectin can also form a double-domain fusion protein via a short linker between two single domains.

Here, we utilize the dynamical network analysis to investigate the structural characteristic of the bulb-type mannose binding protein^29–34^. The network analysis reveals that the polar residues on the surface with high closeness are responsible for the long-range recognition of dimer formation. This observation is further supported by the direct coupling analysis that shows coevolution of these polar residues in dimer complex but not in double-domain construct. Taken together, these results suggest a new scheme to identify critical residues for bulb-type lectin complex formation that may be reengineered for novel biomedical applications.

## Methods

### Molecular dynamics simulations

The MD simulations were carried out using the GROMACS software package^35^. The AMBER03 force field^36^ and TIP3P^37^ water solvation model were used for the simulations. A water solvent box of 12 Å was created between the outside of the protein and the edge of the box. All the structures were simulated at the room temperature (300 K). The initial structure was extracted from the PDB database (PDB code: 1KJ1)^18^ and solvated with water molecules in a periodic rectangular box with a normal saline condition. The SHAKE algorithm was used to constrain all bond lengths^38^. The long-range electrostatic interactions were treated with the Particle Mesh Ewald method^39^. The non-bonded (electrostatic and VDW) cutoff range was 8 Å. A time step of 2 fs was used for numerical integration. Before the MD simulation, the entire system was first minimized by steepest descent calculation for 1000 steps followed by 300 ps equilibration. For each state, three 30 ns trajectories were generated. The solvent accessible surface areas were calculated by GETAREA^40^. The interface area is defined as the accessible surface on each of the two partners that subsequently become inaccessible to solvent in their dimer formation. The structures were visualized and analyzed by VMD and PyMOL^41^.

### Network construction

A dynamical network was constructed by Carma package from the final 20 ns portion of the entire 30 ns trajectories^33, 42^. A node in the network denotes a single amino acid residue. Two nonconsecutive residues in sequence are connected by an edge if they contain a pair of heavy atoms, one from each residue, less than 4.5 Å apart for at least 75% of the times during the MD simulation. The weight of the edge between two connected nodes *i* and *j* is defined as:

1 $${{W}}_{{ij}}=-\mathrm{log}(|{{C}}_{{ij}}|)$$

with *C*_*ij*_ measuring the correlation of motions of nodes *i* and *j*:

2 $${C}_{ij}=\frac{\langle {\rm{\Delta }}{\vec{r}}_{i}(t)\cdot {\rm{\Delta }}{\vec{r}}_{j}(t)\rangle }{{(\langle {\rm{\Delta }}{\vec{r}}_{i}{(t)}^{2}\rangle \langle {\rm{\Delta }}{\vec{r}}_{j}{(t)}^{2}\rangle )}^{1/2}}and\,{\rm{\Delta }}{\vec{r}}_{i}(t)={\vec{r}}_{i}(t)-\langle {\vec{r}}_{i}(t)\rangle $$

where ${\vec{r}}_{i}(t)$ is the position vector of the *C*_*α*_ atom of the *i* ^*th*^ amino acid and the brackets indicate the time average. The values of *C*_*ij*_ vary from −1 to 1. Since we focused on the nodes moving together in the same direction, we removed the edges if their correlations were from −1 to 0.

### Networks analysis

We analyzed the closeness, betweenness, characteristic path length (CPL), and delta path length (DPL) of the coarse-grained dynamical network where only *C*_*α*_ atoms of amino acids are used to construct the network. The closeness of a node is defined as the inverse of the sum of its shortest distances to all other nodes as the following:

3 $$C(x)=\frac{n-1}{{\sum }^{}d(x,y)}$$

where *d*(*x*, *y*) is the distance of the shortest path between the node *x* and any other node *y* ^43^. The betweenness of a node *x* measures its contribution toward the network communication by counting the number of shortest paths between all pairs of nodes that also pass through the node *x*. The CPL is the average length of the shortest paths between all pairs of nodes. The DPL of a node *x* is the change of CPL induced by removing the node *x*. The shortest paths between all pairs of nodes are found using the Floyd-Warshall algorithm.

### Sequence evolution analysis

The sequence evolution analysis measures the residue conservation by ConSurf program^44^. First, we obtained the alignment files (PDB code: 1KJ1, for chain A and chain D) from ConSurf-DB^45^. To focus on the plant lectins, we filtered the sequences by manually removing all non-plant entries. The final numbers of sequences were 79 for chain A (Table S1) and 82 for chain D (Table S2), respectively. Then, we used the program ClustalW2 to perform the sequence alignment on the filtered sequences^46, 47^. Lastly, we calculated the residue conservation scores by ConSurf^44^. The continuous conservation scores are divided into a discrete scale of 9 grades with grade 1–3 for the most variable positions and grade 7–9 for the most conserved positions. The 46 single-domain and 16 double-domain sequences were obtained from the annotations of the homology sequences in UniProt^48^.

### Sequence coevolution analysis

The Direct Coupling Analysis (DCA) was performed to infer the interacting residues by using information on sequence coevolution across different species^49–52^. The single- and double-domain sequence alignments were listed in two files: Supplementary Info File 2 (SI 2) and Supplementary Info File 3 (SI 3). The MUSCLE^53^ program was used to perform the sequence alignment. The main steps of DCA are as follows.

Step 1: the columns in multiple sequence alignment (MSA) showing more than 50% gaps are removed.

Step 2: amino acid frequencies for single residue *f*_*i*_(*A*_*i*_) and a pair of residues *f*_*ij*_(*A*_*i*_, *A*_*j*_) are computed by reweighting the *M* sequences in MSA based on sequence identity as the following:

4 $${f}_{i}({A}_{i})=\frac{1}{\lambda +{M}_{eff}}(\frac{\lambda }{21}+\sum _{a=1}^{M}\frac{1}{{m}_{a}}{\delta }_{{A}_{i},{A}_{i}^{a}})$$

5 $${f}_{ij}({A}_{i},{A}_{j})=\frac{1}{\lambda +{M}_{eff}}(\frac{\lambda }{{21}^{2}}+\sum _{a=1}^{M}\frac{1}{{m}_{a}}{\delta }_{{A}_{i},{A}_{i}^{a}}{\delta }_{{A}_{j},{A}_{j}^{a}})$$

Here *A*_*i*_ (*A*_*j*_) denotes what is at the *i* ^*th*^ *(j* ^*th*^ *)* location of the sequence of length L, which can be one of the 21 possible choices including 20 actual amino acid types or a gap insertion in MSA. A pseudo-count *λ* = 0.5 is introduced to treat possible finite sample effect. ${M}_{eff}=\sum _{a=1}^{M}1/{m}_{a}$ is the effective number of sequences where *m*_*a*_ counts the number of sequences with more than 80% sequence identity to the *a* ^*th*^ sequence $\{{A}_{1}^{a},\,\ldots ,{A}_{L}^{a}\}$ in MSA.

Step 3: the model statistical probabilities of a single residue and a pair of residues in MSA are,

6 $${P}_{i}({A}_{i})=\sum _{\{{A}_{k}|k\ne i\}}P({A}_{1},\ldots ,{A}_{L})$$

7 $${P}_{ij}({A}_{i},{A}_{j})=\sum _{\{{A}_{k}|k\ne i,j\}}P({A}_{1},\ldots ,{A}_{L})$$

where *P*(*A*_1_, …, *A*_*L*_) is the global probability of the sequence {*A*_1_, … *A*_*L*_}. Using the maximum-entropy model, the global probability involves the residue-pair interaction energy (pairwise couplings) *e*_*ij*_(*A*_*i*_, *A*_*j*_) and local energy *h*_*i*_(*A*_*i*_),

8 $$P({A}_{1},\ldots ,{A}_{L})=\exp \{\sum _{i < j}{e}_{ij}({A}_{i},{A}_{j})+\sum _{i}{h}_{i}({A}_{i})\}/Z$$

the normalization factor was defined as $Z=\sum _{{A}_{1},\,\ldots ,\,{A}_{L}}\exp \{\sum _{i < j}{e}_{ij}\,({A}_{i},{A}_{j})+\sum _{i}{h}_{i}({A}_{i})\}$ Applying the mean-field approximation, the residue-pair interaction energy can be estimated by the inverse of the covariance matrix,

9 $${e}_{ij}({A}_{i},{A}_{j})=-{C}_{ij}{({A}_{i},{A}_{j})}^{-1}$$

where the covariance matrix is *C*_*ij*_(*A*_*i*_, *A*_*j*_) = *P*_*ij*_(*A*_*i*_, *A*_*j*_)−*P*_*i*_(*A*_*i*_)*P*_*j*_(*A*_*j*_). Since we want to fit the one-site and two-site marginal of *P*(*A*_1_, …, *A*_*L*_) to the empirical reweighted counting frequency *f*_*i*_(*A*_*i*_) and *f*_*ij*_(*A*_*i*_, *A*_*j*_), we substitute *C*_*ij*_(*A*_*i*_, *A*_*j*_) in the above equation with ${C}_{ij}^{emp}({A}_{i},{A}_{j})=\,{f}_{ij}({A}_{i},{A}_{j})-{f}_{i}({A}_{i}){f}_{j}({A}_{j})$.

Step 4: the direct couplings are defined as

10 $$D{I}_{ij}=\sum _{AB}{P}_{ij}^{d}(A,B)\mathrm{ln}\,\frac{{P}_{ij}^{d}(A,B)}{{f}_{i}(A){f}_{j}(B)}$$

with the help of an isolated two-site model

11 $${P}_{ij}^{d}(A,B)=\exp \{{e}_{ij}(A,B)+{\tilde{h}}_{i}(A)+{\tilde{h}}_{j}(B)\}/{Z}_{ij}$$

${\tilde{h}}_{i}(A)$ and ${\tilde{h}}_{j}(B)$ are defined by the empirical single-residue frequency ${f}_{i}(A)=\sum _{B}{P}_{ij}^{d}(A,B)$ and ${f}_{j}(B)=\sum _{A}{P}_{ij}^{d}(A,B)$.

## Results

### Interface and mannose binding residues are more conserved than other surface residues

**Figure 1:**
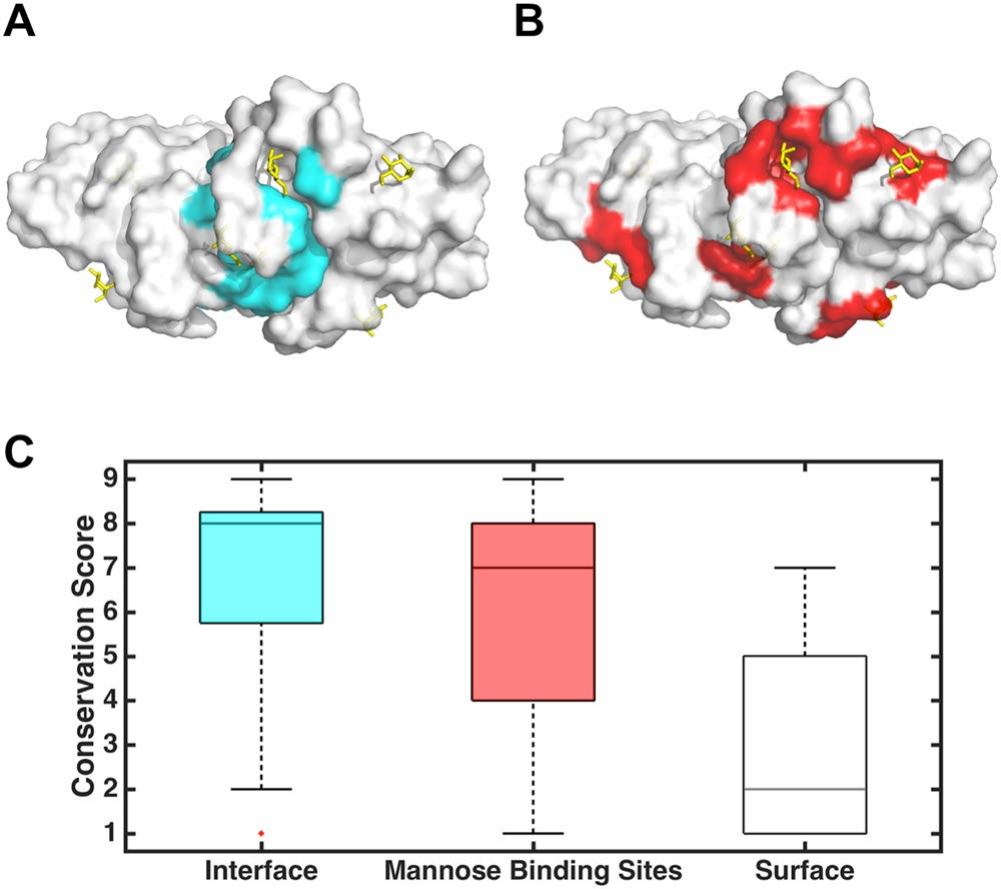
Classification of interface, mannose binding, and surface residues of the garlic mannose-binding lectin protein and their sequence conservation scores. (**A**) and (**B**) represent typical mapping of interface and mannose binding residues on a 3D structure, colored in cyan and red, respectively. (**C**) Distributions of average conservation scores of interface, mannose binding, and surface residues.

The tertiary structure of the garlic bulb-type lectin protein was extracted from PDB database with a resolution of 2.2 Å (PDB code: 1KJ1)^18^. To understand the structural characteristic, we divide the protein into surface sites, interface sites, mannose-binding sites, and interior sites. A residue is defined as interface if it is solvent exposed in monomer but not solvent exposed in dimer complex. The surface residues are the solvent exposed residues both in monomer and dimer complex (Fig. 1A, Table S3). The solvent accessible surface area of a residue was calculated using the solvent accessible surface recognition program GETAREA (Table S5)^40^. The mannose binding sites were identified by protein-ligand interaction recognition program LIGPLOT^54^ (Fig. 1B, Table S4, Figure S2). We then performed sequence evolution analysis to investigate the sequence conservation of surface, interface, and mannose-binding residues in the dimer complex, respectively (see Materials and Methods for details). As shown in Fig. 1C, interface sites (average conservation score = 6.72, standard deviation = 2.46) and mannose binding sites (average conservation score = 6.18, standard deviation = 2.35) are significantly more conserved than the surface sites (average conservation score = 2.84, standard deviation = 1.90). It is believed that the interface residues tend to be more conserved across lectins for the stability of dimer formation while the slightly more varied mannose binding sites are for different mannose-binding specificity.

Table 1 lists all interacting pairs of the interface. A pair of amino acids across the interface is defined as interacting if the distance of any two heavy atoms, one from each amino acid, is less than 4 Å. As shown in Table 1, most interacting pairs are fairly conserved to maintain the interface stability. However, there is a highly variable pair of polar residues (A:GLU91-D:MET5). Ref. 53 already observed similar phenomena and suggested that such charged pairs should be for long-range steering effect in dimer formation. As discussed further below, a closeness score may provide a quantitative and practical measure to identify this polar pair. Moreover, our finding shows that this polar pair is largely absent in the double-domain fusion proteins. This offers the clearest evidence yet so far in support of this long-range recognition conjecture.

**Table 1:** Tertiary interactions within 4 Å at the interface. The interactions were calculated from the garlic mannose-binding lectin crystal structure (PDB code: 1KJ1).

Chain A		Chain D		Distance (Å)
Residue	Conservation	Residue	Conservation	
GLU91	2	MET5	1	2.97
ASN94	7	THR105	9	3.34
ASN94	7	THR107	9	2.87
TYR98	8	TYR98	9	3.74
TYR98	8	ASP101	6	3.49
GLY99	7	GLY99	7	2.93
GLY99	7	ILE102	5	3.57
ASP101	6	TYR98	9	3.96
ILE102	5	GLY99	7	3.55
SER104	8	ASN94	8	3.99
THR105	9	ASN94	8	3.17
THR107	9	ASP92	8	3.80
THR107	9	ASN94	8	2.96

### Network analysis reveals the critical residues for intermolecular communication

The interface residues listed in Table 1 were obtained from the static structure of the dimer complex. It is necessary, however, to go beyond the static configuration to probe the importance of these residues in coordinating the dynamical motion of the entire complex. To this end, we performed the MD simulations and used the simulation trajectories of the complex to construct the dynamical network (see Materials and Methods for details). Given the network, graph theory concepts such as betweeness and delta path length (DPL) can be used to quantify the relative importance of each residue for the network communication between two monomers including some subtle allosteric effect.

**Figure 2:**
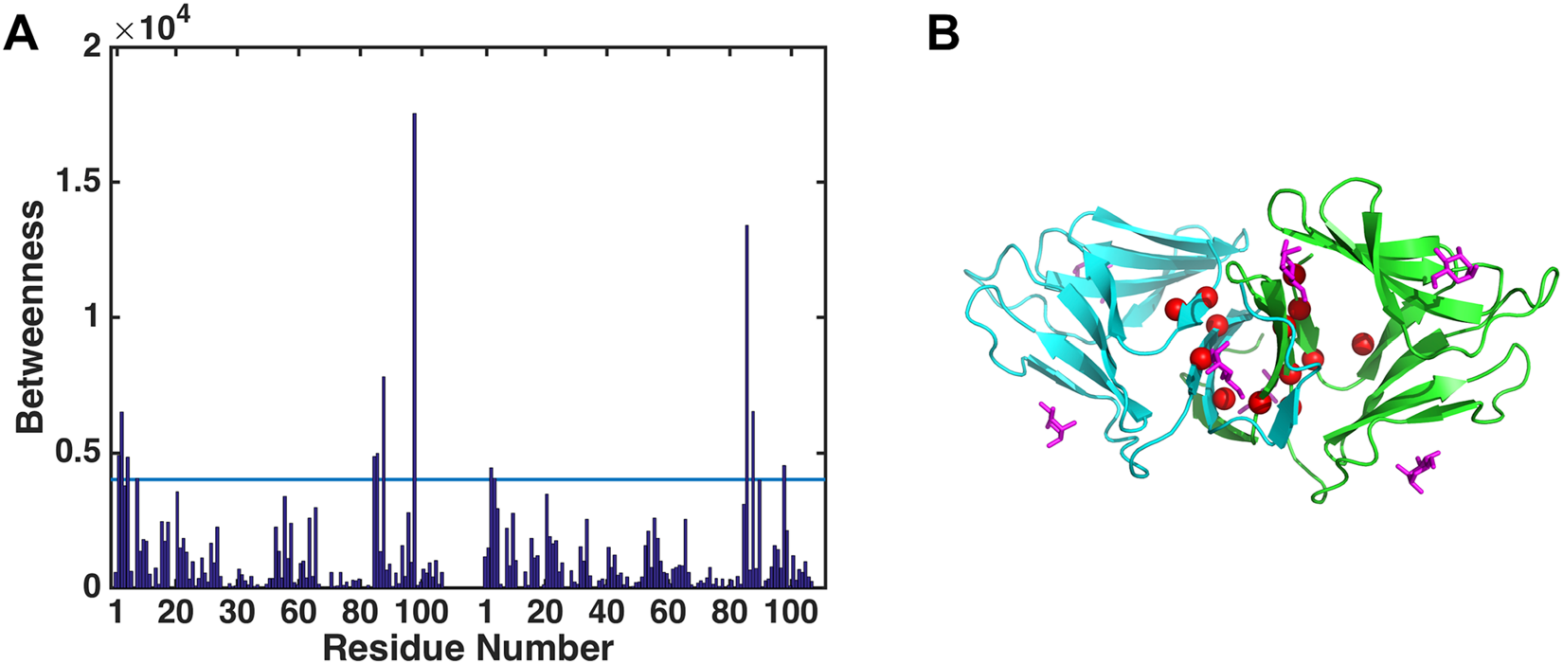
Betweenness centrality of residues in dynamical network. (**A**) ASN2, LEU3, THR5, GLU8, TYR85, VAL86, VAL88, TYR98 of chain A, and ILE3, LEU4, VAL86, VAL88, TYR98 of chain D have large betweenness with a Z-score greater than a cutoff value of 1.5 (light blue line). (**B**) The locations of the significant betweenness residues (red spheres). The chain A, chain D, and mannoses are colored in green, cyan, and magenta, respectively. The TYR98 with highest betweenness is the critical residue for intermolecular network communication.

First, we performed the betweenness calculation of the dynamic network. To probe the communication in this dynamical network, we identified the shortest path between each and every pair of nodes in the network and defined the betweenness of a node as the number of such shortest paths going through the node. Figure 2 shows the betweenness values in the entire dynamical network of the dimer complex with a Z-score value larger than 1.5. Most of the residues of high betweenness, especially TYR98, are located near interface indicating the importance of these residues in maintaining the correlated dynamics of the dimer complex. As such, it is no surprise that these residues are very conserved with conservation scores higher than 6.

**Figure 3:**
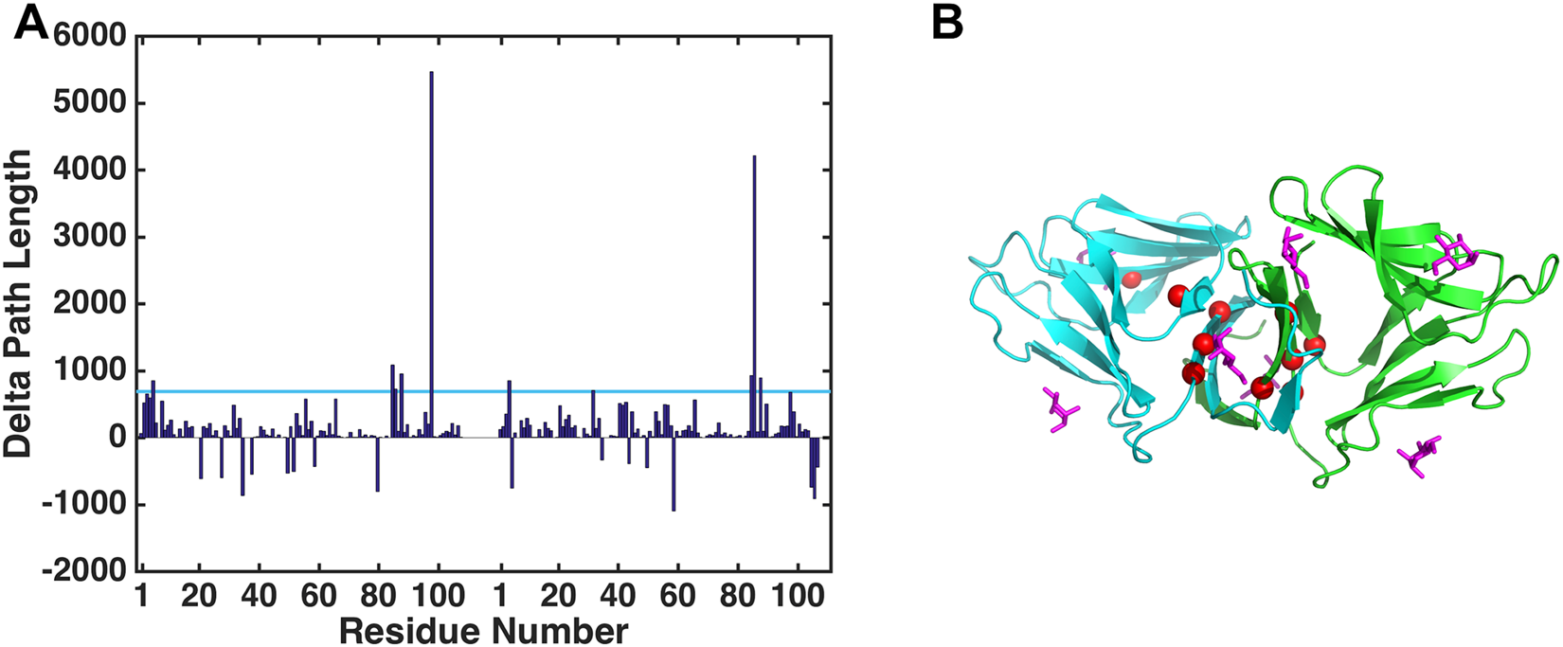
Delta path length of residues in dynamical network. (**A**) THR5, TYR85, VAL86, VAL88, TYR98 of chain A, and LEU4, VAL32, TYR85, VAL86, VAL88 of chain D have large delta path length with a Z-score greater than a cutoff value of 1.0 (light blue line). (**B**) The locations of the significant delta path length residues (red spheres). The chain A, chain D, and mannoses are colored in green, cyan, and magentas, respectively. The TYR98 with highest DPL is the critical residue for intermolecular network communication.

Second, we performed the delta path length (DPL) calculation of the dynamical network. Since betweenness only considers the shortest path, it may overestimate the importance of a node in the network communication where there exist other paths of comparable length such as a very close but distinctive second shortest path. To overcome this potential pitfall, we also computed DPL of a node as the change of the average path length upon removal of the node (see Materials and Method). Figure 3 shows that most residues increase the path length upon their removal from the dynamic network. The values of betweenness and DPL share a high correlation of 0.845. The combined results of both metrics of betweenness and DPL suggest that the highly-conserved residue TYR98 is the critical residue for intermolecular network communication and dimer stability.

### Closeness analysis reveals the critical residues for long-range recognition

In a connected graph, a node with small total distance to all other nodes acts as a hub for inter-network communication. To be precise, the closeness of a node is defined as the inverse of the sum of its shortest distances to all other nodes. It was proposed that the residues of high closeness are functional sites since, as network hubs, they can interact effectively with all other residues either directly or through a few intermediates. Indeed, previous benchmark tests showed that closeness scores successfully identified 70% of the protein active sites^43^.

**Figure 4:**
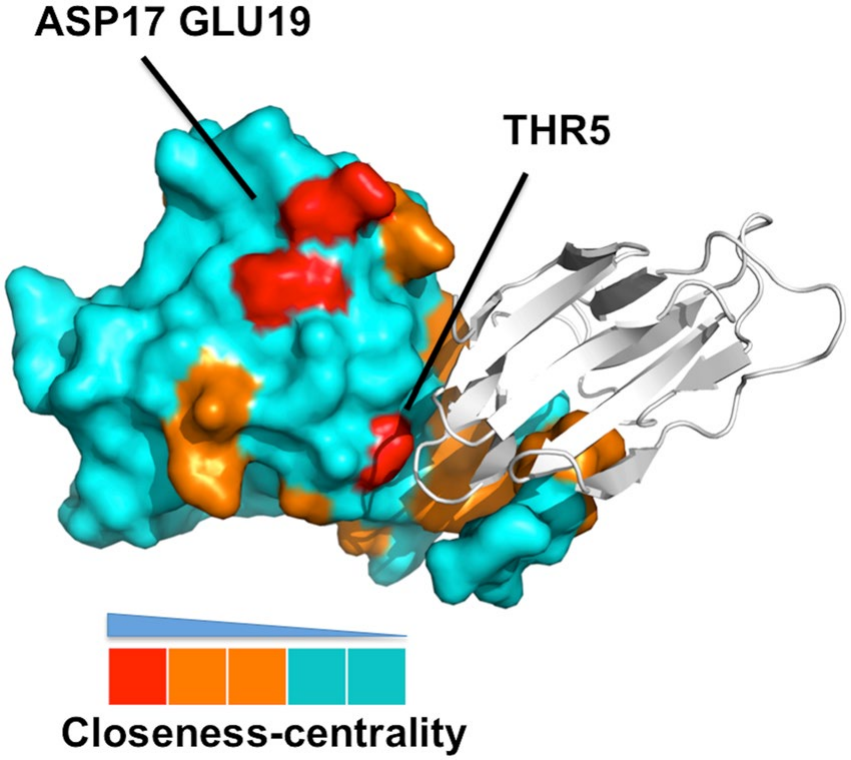
Surface representation of the garlic mannose-binding lectin protein (monomer, chain A). The residues are colored by closeness values with red, orange and cyan corresponding to the high (top 20%), intermediate (20~60%) and low (below than 60%) closeness values. The crystal structure of garlic lectin showed that THR5 is responsible for dimer formation and snowdrop lectin indicated that ASP17/GLU19 might be responsible for tetramer formation.

We further hypothesize that a pair of charged surface residues of high individual closeness value could best exert the long-range steering effect for dimer formation. Since each such residue forms a tighter connection with its own monomer, an attractive interaction for the pair can bring the two monomers together more effectively. To check this, we constructed the dynamic network from the MD simulations of the lectin monomer, and then computed the closeness values of all surface residues and classified them into three categories: (1) most likely recognition sites (high closeness values), (2) likely recognition sites (intermediate closeness values), and (3) unlikely recognition sites (small closeness values). Our results suggested that some residues (THR5, ASP17, and GLU19, colored in red) might be considered as the most likely recognition sites (Fig. 4). The crystal structure of garlic lectin showed that THR5 is responsible for dimer formation^18^, and the crystal structure of snowdrop lectin indicated that ASP17/GLU19 might be responsible for tetramer formation^55^. Therefore, structural information on both monomer and complex and their associated network analysis supported our hypothesis that high closeness polar residues on surface might be responsible for long-range protein-protein recognition.

We analyzed additional representative plant homology sequences in both single- and double-domains of this specific kind of lectin (Supplementary Info 2 and 3). There are four such lectins with known crystal structures (Table S7)^56–58^. We compared the differences between single- and double-domain lectin proteins: (1) both single-domain lectin protein structures (PDB code: 1MSA and 3A0C) are similar to garlic lectin (PDB code: 1KJ1) with RMSDs around 1.5 Å, while double-domain protein structures (PDB code: 3MEZ and 3R0E) are different with RMSDs larger than 3 Å; (2) the residue-residue interaction of position 5 and position 91 is polar-polar interaction in single-domain lectin proteins but not in double-domain proteins; (3) the residues of position 5 and position 91 are surface residues in single-domain lectin proteins but not in double-domain lectin proteins; and (4) the residue closeness of position 5 is significantly high in single-domain but not in double-domain. These results supported our hypothesis that high closeness polar residues on surface of complex may responsible for long-range protein-protein recognition.

### Direct coupling analysis reveals that the polar pair is coevolving

While it is possible to verify the importance of some residues toward the dimer formation via site mutagenesis, it is not clear how to measure if they are for short-range or long-range effect. Fortunately, the abundance of single- and double-domain lectins in plant genomes offers a unique opportunity to verify our hypothesis on long-range recognition mechanism. Unlike the dimeric lectin complex formed via protein-protein interaction of two single-domain lectins, the double-domain lectin is formed by fusing two single-domain lectins into one protein with a short linker and its two single domains are always in close proximity and thus do not require long-range recognition for its formation. Therefore, sequence features present in the single domain but absent in the double domain may be attributed to long-range effect. Specifically, we performed hydrophobic-polar pattern analysis and sequence coevolution analysis for both single- and double-domain lectins at position 91 and 5 to probe GLU91-MET5 pair.

**Figure 5:**
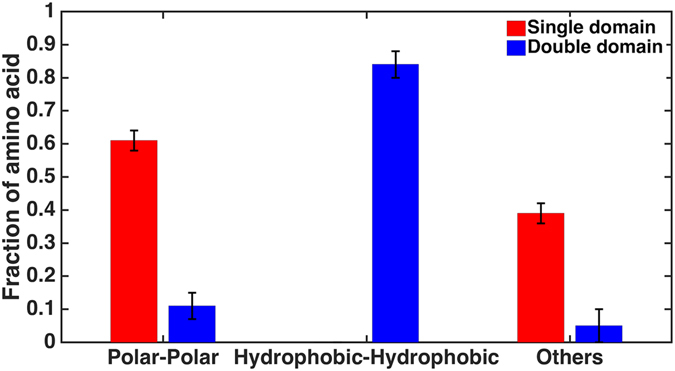
The analysis of interaction pattern between position 5 and 91 for single-domain and double-domain lectins in plant genomes. The red and blue colors are for the single-domain and double-domain lectins, respectively. We randomly select about 31 out of 46 single-domain sequences and 11 out of 16 double-domain sequences and analyze their interaction pattern, respectively. This is repeated five times. The residues at position 5 and 91 prefer to form polar-polar interaction pairs in single-domain lectins but hydrophobic-hydrophobic interaction pairs in double-domain lectins.

For the hydrophobic-polar pattern analysis, we count the number of different types of interactions. In order to analyze the interaction pattern statistically, we randomly select 31 out of 46 single-domain sequences and 11 out of 16 double-domain sequences to compare the interaction patterns for single- and double-domain lectins, respectively. This calculation is repeated five times. The results indicate that the residues at position 5 and 91 prefer to form polar-polar interaction pairs in single-domain lectins but hydrophobic-hydrophobic interaction pairs in double-domain lectins. Figure 5 shows that dimer complex prefers polar interactions while such charged pair of GLU91-MET5 is largely absent in the double-domain lectins. This is strong evidence that GLU91-MET5 is indeed for long-range steering effect.

Direct Coupling Analysis (DCA) uses a global statistical model of multiple sequence alignments to infer direct interaction from coevolution of residue pairs^51, 59, 60^. We performed DCA for both single and double domains to identify the coevolving patterns for those residue pairs as displayed in Table 1. In previous coevolution analysis, the number of sequences used was typically comparable to the length of the target protein, and it was found that a DI score of 0.8 indicates a significant co-evolutionary signal^61, 62^. Therefore, we focused on the 18 residues at interface to perform the coevolution calculation due to the limited sequence information. The results indicate that less conserved residue pairs (with conservation scores less than 8 for both residues in the pair, Table S6) are coevolving in single-domain (with DI score greater than 0.8) but not in double-domain (with DI score less than 0.8). Specifically, the DI scores for GLU91-MET5 pair shows that there is strong correlation between GLU91 and MET5. The GLU91-MET5 pair is coevolved to maintain the interaction for long-range recognition in single-domain.

## Discussion and Conclusion

Protein-protein interactions are essential for carrying out various biological functions. Previous large-scale analysis of protein-protein interface of known complexes discovered a surprising pattern of highly variable and charged residue pairs at the interface. It was suggested that these pairs might provide the long-range steering force to bring together interacting proteins for dimer formation. Indeed, some mutagenesis experiments confirmed the importance of these charged pairs on protein surface for dimer formation and binding specificity^63–65^.

The results from our case study on mannose-binding lectin complex are also consistent with this hypothesis. But beyond the qualitative description, we further proposed three practical and quantitative metrics to pinpoint such charged pairs for long-range recognition among a multitude of charged residues on protein surface without the complete structure of the dimer complex. Specifically, these charged pairs have the following unique features: (1) high closeness in the dynamical network of the monomer; (2) strong direct coupling indicating coevolution in the multiple sequence alignment; and (3) its absence in the double-domain construct. The last two measures above require sequence analysis only.

The identification of critical residue pairs for complex formation has many benefits. These pairs can serve as distance constraints to guide the structure modeling for much better accuracy. They can also facilitate drug design or protein engineering in order to regulate the complex formation for biological or medical applications.

In summary, we developed a hybrid approach of structure modeling, network analysis, and sequence statistical inference to identify critical residues for protein complex formation. Our results suggest that the coevolving polar residue pairs of high closeness initiate the long-range recognition of the bulb-type lectin complex formation that is further stabilized by short-range complementary interactions.

## Electronic supplementary material

Supplementary Info
