# Supplementary material for: Network Analysis Reveals the Recognition Mechanism for Dimer Formation of Bulb-type Lectins
Source: Sci Rep. 2017 Jun 6;7:2876. doi: 10.1038/s41598-017-03003-5 (PMC5460271; doi:10.1038/s41598-017-03003-5)
Supplement: Supplementary file 1 — Supplementary Info [file 41598_2017_3003_MOESM1_ESM.pdf]

Supplementary Info

Network Analysis Reveals the Recognition Mechanism for Dimer Formation of Bulb-type Lectins

Yunjie Zhao[1,3†], Yiren Jian[3†], Zhichao Liu[3], Hang Liu[4], Qin Liu[5], Chanyou Chen[5], Zhangyong Li[2], Lu Wang[2], H. Howie Huang[4] and Chen Zeng[2,3,5*]

Author affiliation:
[1] Institute of Biophysics and Department of Physics, Central China Normal University, Wuhan 430079, China
[2] Research Center of Biomedical Engineering, Chongqing University of Posts and Telecommunications, Chongqing, China
[3] Department of Physics, The George Washington University, Washington, DC 20052, USA.
[4] Department of Electrical and Computer Engineering, The George Washington University, Washington, DC 20052, USA.
[5] School of Life Sciences, Jianghan University, Wuhan 430056, China.

**Corresponding authors*
Chen Zeng
Email: chenz@gwu.edu *Phone: +1-202-994-6481, Fax: +1-202-994-3001*

†These authors contributed equally to the paper as first authors.

Table S1. Representative plant homology sequences of chain A.

Species	UniRef ID	Pfam	SMART
Allium	UniRef90_E7CG22_29_137	PF01453	SM00108
Allium cepa	UniRef90_Q679P2_1_109	PF01453	SM00108
Allium sativum	UniRef90_Q38787_37_144	PF01453	SM00108
Allium sativum	UniRef90_Q38787_187_295	PF01453	SM00108
Allium sativum	UniRef90_P92932_155_260	PF01453	SM00108
Allium sativum	UniRef90_P92932_13_125	PF01453	SM00108
Allium sativum	UniRef90_Q38783_29_137	PF01453	SM00108
Allium triquetrum	UniRef90_Q0R369_29_138	PF01453	SM00108
Allium ursinum	UniRef90_Q38725_28_135	PF01453	SM00108
Allium ursinum	UniRef90_O24427_29_137	PF01453	SM00108
Allium ursinum	UniRef90_Q38724_22_128	PF01453	SM00108
Alocasia macrorrhizos	UniRef90_Q2LAE3_27_125	PF01453	SM00108
Alocasia macrorrhizos	UniRef90_Q2LAE3_146_247	PF01453	SM00108
Aloe arborescens	UniRef90_P49329_2_107	PF01453	SM00108
Amaryllidoideae	UniRef90_Q7Y1W7_25_130	PF01453	SM00108
Amaryllis minuta	UniRef90_Q6XCI6_27_134	PF01453	SM00108
Ananas comosus	UniRef90_Q8H2A4_24_129	PF01453	SM00108
Arisaema amurense	UniRef90_B1A014_147_248	PF01453	SM00108
Arisaema amurense	UniRef90_B1A014_28_126	PF01453	SM00108
Arisaema lobatum	UniRef90_Q6Q5X6_27_121	PF01453	SM00108
Arisaema lobatum	UniRef90_Q6Q5X6_148_250	PF01453	SM00108
Arisaemateae	UniRef90_E2ITD6_149_250	PF01453	SM00108
Arisaemateae	UniRef90_E2ITD6_28_122	PF01453	SM00108
Arum maculatum	UniRef90_Q38731_148_251	PF01453	SM00108
Arum maculatum	UniRef90_Q38731_28_122	PF01453	SM00108
Bulbophyllum morphologorum	UniRef90_F8SK84_30_134	PF01453	SM00108
Chimonanthus praecox	UniRef90_A2SVT1_39_144	PF01453	SM00108
Clivia miniata	UniRef90_Q39542_32_136	PF01453	SM00108
Clivia miniata	UniRef90_Q39544_29_136	PF01453	SM00108
Crinum asiaticum	UniRef90_Q6SQE5_24_131	PF01453	SM00108
Crocus	UniRef90_Q9FVA1_27_131	PF01453	SM00108
Crocus	UniRef90_Q9FVA1_150_255	PF01453	SM00108
Crocus vernus	UniRef90_P86626_117_222	PF01453	SM00108
Cymbidium hybrid cultivar	UniRef90_Q39512_31_135	PF01453	SM00108
Dendrobium	UniRef90_B2KNH9_26_135	PF01453	SM00108
Dioscorea polystachya	UniRef90_Q5W956_5_108	PF01453	SM00108
Epipactis helleborine	UniRef90_Q39728_27_131	PF01453	SM00108
Epipactis helleborine	UniRef90_Q39729_32_136	PF01453	SM00108
Galanthus nivalis	UniRef90_P30617_25_129	PF01453	SM00108
Galanthus nivalis	UniRef90_Q39904_21_128	PF01453	SM00108
Galanthus nivalis	UniRef90_Q39903_24_128	PF01453	SM00108

Galanthus nivalis	UniRef90_Q945J7_24_129	PF01453	SM00108
Hernandia moerenhoutiana subsp. samoensis	UniRef90_Q84RN0_1_93	PF01453	SM00108
Hippeastrum	UniRef90_Q7Y041_26_133	PF01453	SM00108
Hippeastrum sp.	UniRef90_Q39991_1_84	PF01453	SM00108
Hyacinthoides hispanica	UniRef90_Q9ZP48_38_133	PF01453	SM00108
Hyacinthoides hispanica	UniRef90_Q9ZP49_53_138	PF01453	SM00108
Hyacinthoides hispanica	UniRef90_Q9ZP48_162_258	PF01453	SM00108
Lycoris radiata	UniRef90_Q4W660_23_130	PF01453	SM00108
Lycoris sp.	UniRef90_Q5MK12_24_129	PF01453	SM00108
Lysichiton camtschatcensis	UniRef90_H1A7C5_27_126	PF01453	SM00108
Lysichiton camtschatcensis	UniRef90_H1A7C5_155_255	PF01453	SM00108
Magnoliophyta	UniRef90_B4XS32_3_111	PF01453	SM00108
Monstera deliciosa	UniRef90_A2IBI1_176_263	PF01453	SM00108
Monstera deliciosa	UniRef90_A2IBI1_32_142	PF01453	SM00108
Narcissus hybrid cultivar	UniRef90_Q40417_26_132	PF01453	SM00108
Narcissus hybrid cultivar	UniRef90_Q40423_1_82	PF01453	SM00108
Narcissus tazetta	UniRef90_C9W8B3_27_133	PF01453	SM00108
Pandanus amaryllifolius	UniRef90_F8UZX3_32_132	PF01453	SM00108
Pinellia	UniRef90_D4P965_28_121	PF01453	SM00108
Pinellia	UniRef90_D4P965_147_248	PF01453	SM00108
Pinellia ternata	UniRef90_H8Y197_148_249	PF01453	SM00108
Pinellia ternata	UniRef90_H8Y197_28_121	PF01453	SM00108
Polygonatum cyrtonema	UniRef90_Q8L568_30_135	PF01453	SM00108
Polygonatum multiflorum	UniRef90_O24274_31_135	PF01453	SM00108
Polygonatum multiflorum	UniRef90_O24273_33_128	PF01453	SM00108
Polygonatum roseum	UniRef90_Q5EER7_30_134	PF01453	SM00108
Remusatia vivipara	UniRef90_B5LYJ9_146_247	PF01453	SM00108
Remusatia vivipara	UniRef90_B5LYJ9_29_120	PF01453	SM00108
Taxus x media	UniRef90_Q56V23_27_136	PF01453	SM00108
Tulipa hybrid cultivar	UniRef90_Q41625_51_155	PF01453	SM00108
Tulipa hybrid cultivar	UniRef90_Q41622_21_126	PF01453	SM00108
Typhonium divaricatum	UniRef90_Q6V8L5_36_141	PF01453	SM00108
Typhonium divaricatum	UniRef90_A2ICN3_28_121	PF01453	SM00108
Typhonium divaricatum	UniRef90_A2ICN3_147_250	PF01453	SM00108
Zantedeschia aethiopica	UniRef90_Q6JTD2_25_130	PF01453	SM00108
Zephyranthes candida	UniRef90_Q8LK14_28_134	PF01453	SM00108
Zingiber officinale	UniRef90_B7U6V0_23_126	PF01453	SM00108

Table S2. Representative plant homology sequences of chain D.

Species	UniRef ID	Pfam	SMART
Allium	UniRef90_E7CG22_29_137	PF01453	SM00108
Allium cepa	UniRef90_Q679P2_1_109	PF01453	SM00108
Allium sativum	UniRef90_Q38787_187_295	PF01453	SM00108
Allium sativum	UniRef90_Q38787_37_144	PF01453	SM00108
Allium sativum	UniRef90_P92932_155_260	PF01453	SM00108
Allium sativum	UniRef90_P92932_13_125	PF01453	SM00108
Allium triquetrum	UniRef90_Q0R369_29_138	PF01453	SM00108
Allium ursinum	UniRef90_Q38725_28_135	PF01453	SM00108
Allium ursinum	UniRef90_O24427_29_137	PF01453	SM00108
Allium ursinum	UniRef90_Q38724_22_128	PF01453	SM00108
Alocasia macrorrhizos	UniRef90_Q2LAE3_146_249	PF01453	SM00108
Alocasia macrorrhizos	UniRef90_Q2LAE3_27_129	PF01453	SM00108
Aloe arborescens	UniRef90_P49329_2_107	PF01453	SM00108
Amaryllidoideae	UniRef90_Q7Y1W7_25_130	PF01453	SM00108
Amaryllis minuta	UniRef90_Q6XCI6_27_134	PF01453	SM00108
Ananas comosus	UniRef90_Q8H2A4_24_129	PF01453	SM00108
Arisaema amurense	UniRef90_B1A014_147_250	PF01453	SM00108
Arisaema amurense	UniRef90_B1A014_25_130	PF01453	SM00108
Arisaema lobatum	UniRef90_Q6Q5X6_27_130	PF01453	SM00108
Arisaema lobatum	UniRef90_Q6Q5X6_148_252	PF01453	SM00108
Arisaemateae	UniRef90_E2ITD6_149_252	PF01453	SM00108
Arisaemateae	UniRef90_E2ITD6_28_131	PF01453	SM00108
Arum maculatum	UniRef90_Q38731_28_129	PF01453	SM00108
Arum maculatum	UniRef90_Q38731_148_253	PF01453	SM00108
Bulbophyllum morphologorum	UniRef90_F8SK84_30_134	PF01453	SM00108
Chimonanthus praecox	UniRef90_A2SVT1_39_144	PF01453	SM00108
Clivia miniata	UniRef90_Q39542_32_136	PF01453	SM00108
Clivia miniata	UniRef90_Q39544_29_135	PF01453	SM00108
Crinum asiaticum	UniRef90_Q6SQE5_24_131	PF01453	SM00108
Crocus	UniRef90_Q9FVA1_150_255	PF01453	SM00108
Crocus	UniRef90_Q9FVA1_27_131	PF01453	SM00108
Crocus vernus	UniRef90_P86626_117_222	PF01453	SM00108
Cymbidium hybrid cultivar	UniRef90_Q39512_31_135	PF01453	SM00108
Dendrobium	UniRef90_B2KNH9_26_135	PF01453	SM00108
Dioscorea polystachya	UniRef90_Q5W956_5_108	PF01453	SM00108
Epipactis helleborine	UniRef90_Q39728_27_131	PF01453	SM00108
Epipactis helleborine	UniRef90_Q39729_40_136	PF01453	SM00108
Galanthus nivalis	UniRef90_P30617_25_129	PF01453	SM00108
Galanthus nivalis	UniRef90_Q39903_24_128	PF01453	SM00108
Galanthus nivalis	UniRef90_Q39904_21_127	PF01453	SM00108
Galanthus nivalis	UniRef90_Q945J7_24_129	PF01453	SM00108

Hernandia moerenhoutiana subsp. samoensis	UniRef90_Q84RN0_1_93	PF01453	SM00108
Hippeastrum	UniRef90_Q7Y041_26_132	PF01453	SM00108
Hippeastrum sp.	UniRef90_Q39991_1_83	PF01453	SM00108
Hyacinthoides hispanica	UniRef90_Q9ZP48_38_133	PF01453	SM00108
Hyacinthoides hispanica	UniRef90_Q9ZP49_53_140	PF01453	SM00108
Hyacinthoides hispanica	UniRef90_Q9ZP48_162_258	PF01453	SM00108
Listera ovata	UniRef90_Q40233_29_134	PF01453	SM00108
Listera ovata	UniRef90_Q40231_31_136	PF01453	SM00108
Listera ovata	UniRef90_Q40232_39_135	PF01453	SM00108
Lycoris radiata	UniRef90_Q4W660_23_129	PF01453	SM00108
Lycoris sp.	UniRef90_Q5MK12_24_129	PF01453	SM00108
Lysichiton camtschatcensis	UniRef90_H1A7C5_27_125	PF01453	SM00108
Lysichiton camtschatcensis	UniRef90_H1A7C5_155_257	PF01453	SM00108
Magnoliophyta	UniRef90_B4XS32_3_111	PF01453	SM00108
Monstera deliciosa	UniRef90_A2IBI1_155_263	PF01453	SM00108
Monstera deliciosa	UniRef90_A2IBI1_32_142	PF01453	SM00108
Narcissus hybrid cultivar	UniRef90_Q40423_1_82	PF01453	SM00108
Narcissus hybrid cultivar	UniRef90_Q40417_26_131	PF01453	SM00108
Narcissus tazetta	UniRef90_C9W8B3_27_132	PF01453	SM00108
Pandanus amaryllifolius	UniRef90_F8UZX3_30_132	PF01453	SM00108
Pinellia	UniRef90_D4P965_30_130	PF01453	SM00108
Pinellia	UniRef90_D4P965_147_250	PF01453	SM00108
Pinellia ternata	UniRef90_H8Y197_148_251	PF01453	SM00108
Pinellia ternata	UniRef90_H8Y197_28_130	PF01453	SM00108
Polygonatum cyrtonema	UniRef90_Q8L568_30_135	PF01453	SM00108
Polygonatum multiflorum	UniRef90_O24274_31_135	PF01453	SM00108
Polygonatum roseum	UniRef90_Q5EER7_30_134	PF01453	SM00108
Remusatia vivipara	UniRef90_B5LYJ9_146_249	PF01453	SM00108
Remusatia vivipara	UniRef90_B5LYJ9_29_129	PF01453	SM00108
Taxus x media	UniRef90_Q56V23_27_136	PF01453	SM00108
Tulipa hybrid cultivar	UniRef90_Q41625_51_155	PF01453	SM00108
Tulipa hybrid cultivar	UniRef90_Q41622_21_128	PF01453	SM00108
Typhonium divaricatum	UniRef90_Q6V8L5_36_141	PF01453	SM00108
Typhonium divaricatum	UniRef90_A2ICN3_28_130	PF01453	SM00108
Typhonium divaricatum	UniRef90_A2ICN3_147_250	PF01453	SM00108
Wolffia arrhiza	UniRef90_E2ICK0_24_129	PF01453	SM00108
Wolffia australiana	UniRef90_F8SVT9_25_133	PF01453	SM00108
Zantedeschia aethiopica	UniRef90_Q6JTD2_25_130	PF01453	SM00108
Zephyranthes candida	UniRef90_Q8LK14_28_133	PF01453	SM00108
Zingiber officinale	UniRef90_B7U6V0_23_126	PF01453	SM00108

Table S3. Interface residues on the lectin protein dimer structure (PDB code: 1KJ1). The interface area was defined by the area of the accessible surface on both partners that becomes inaccessible to solvent due to protein-protein interactions. The conservation scores were calculated by ConSurf program.

Chain	Residue	Conservation	Chain	Residue	Conservation
A	THR5	1	D	ASN2	9
A	VAL40	4	D	MET5	1
A	GLU91	2	D	ASP92	8
A	ASP92	8	D	ASN94	8
A	ASN94	7	D	TYR98	9
A	TYR98	8	D	GLY99	8
A	GLY99	8	D	ASP101	7
A	ASP101	6	D	ILE102	5
A	ILE102	5	D	TRP103	7
A	TRP103	6	D	SER104	7
A	SER104	8	D	THR105	9
A	THR105	9	D	THR107	9
A	THR107	9			

Table S4. Mannose specific binding residues on the lectin protein structure (PDB code: 1KJ1). The mannose binding residues were calculated by protein-ligand interaction recognition program LIGPLOT. The conservation scores were calculated by ConSurf program.

Mannose	Chain	Residue	Conservation	Mannose	Chain	Residue	Conservation
MAN301	A	ASP60	5	MAN300	A	GLN90	8
MAN301	A	ASN62	7	MAN300	A	ASP92*	8
MAN301	A	TYR66	3	MAN300	A	ASN94*	7
MAN301	A	HIS77	4	MAN300	A	TYR98*	8
MAN301	A	VAL79	1	MAN300	D	ASN84	4
MAN302	A	GLN26	8	MAN300	D	ASP101*	7
MAN302	A	ASP28	9	MAN303	A	TYR21	5
MAN302	A	ASN30	9	MAN303	A	ASP35	2
MAN302	A	TYR34	8	MAN303	A	VAL40	4
MAN302	A	SER39	1	MAN303	A	GLY93	6
MAN305	D	GLN58	7	MAN303	D	THR107*	9
MAN305	D	ASP60	5	MAN303	D	LYS109	5
MAN305	D	ASN62	7	MAN304	A	ASN84	4
MAN305	D	TYR66	4	MAN304	A	ASP101*	6
MAN305	D	SER72	3	MAN304	A	TYR108	6
MAN306	D	GLN26	8	MAN304	D	GLN90	8
MAN306	D	ASP28	9	MAN304	D	ASP92*	8
MAN306	D	ASN30	9	MAN304	D	ASN94*	8
MAN306	D	TYR34	8	MAN304	D	TYR98*	9
MAN306	D	ASP46	4				

The mannose (MAN) specific binding residues are identified by using LigPlus.
The left column shows the mannose interacts with only one chain (Chain A or Chain D).
The right column shows the mannose interacts with both chain A and Chain D.
The residues at interface area are labeled with *.

Table S5. Surface residues on the lectin protein dimer structure (PDB code: 1KJ1). The solvent accessible surface areas were calculated by GETAREA. The conservation scores were calculated by ConSurf program.

Chain	Residue	Conservation	Chain	Residue	Conservation
A	GLY7	5	D	ASP7	5
A	TYR11	1	D	TYR11	1
A	GLY13	5	D	GLY13	6
A	GLN14	4	D	GLU19	2
A	ASP17	1	D	GLU27	1
A	GLU19	2	D	HIS36	1
A	GLU27	1	D	SER37	5
A	HIS36	1	D	THR38	1
A	SER37	3	D	ALA39	3
A	THR38	1	D	ASN44	7
A	ASN44	6	D	ILE47	6
A	ILE47	6	D	PRO48	1
A	LEU48	1	D	GLY49	6
A	GLY49	5	D	LYS51	2
A	LYS51	1	D	ALA68	1
A	SER59	3	D	GLU69	1
A	ALA68	1	D	GLY70	1
A	GLU69	1	D	ARG71	1
A	GLY70	5	D	HIS77	3
A	ARG71	1	D	VAL79	1
A	SER72	2	D	ARG80	3
A	ARG80	4	D	ASN82	2
A	ASN82	3	D	GLU91	3
A	SER100	2	D	ASN106	4
A	GLY106	5	D	TYR108	3
A	LYS109	5			

Table S6. The direct coupling analysis scores of the interface interactions.

Chain A		Chain D		DI	DI
Residue	Conservation	Residue	Conservation	(single domain)	(double domain)
GLU91	2	MET5	1	0.8	0.6
ASN94	7	THR105	9	0.2	0.1
ASN94	7	THR107	9	0.2	0.5
TYR98	8	TYR98	9	0.8	0.3
TYR98	8	ASP101	6	0.1	0.1
GLY99	7	GLY99	7	1.37	0.6
GLY99	7	ILE102	5	0.3	0.1
ASP101	6	TYR98	9	0.1	0.1
ILE102	5	GLY99	7	0.3	0.2
SER104	8	ASN94	8	0.1	0.3
THR105	9	ASN94	8	0.1	0.7
THR107	9	ASP92	8	0.1	0.1
THR107	9	ASN94	8	0.1	0.3

Table S7. The comparison analysis between single- and double-domain lectin proteins. The residues of position 5 and 91 are important for interaction in single-domain proteins.

PDB	Organism	Single/Double	Position 5	Surface residue	Position 91	Surface residue	RMSD (Å)
1MSA	Snowdrop	Single	TYR	+	LYS	+	1.42
3A0C	Polygonatum cyrtonema	Single	SER	+	GLN	+	1.65
3MEZ	Crocus vernus	Double	LEU	-	PRO	-	3.17
3R0E	Abrus precatorius	Double	TYR	-	PRO	-	5.77

"+" surface residue
"-" not surface residue
Hydrophobic residues are highlighted in red
RMSDs were calculated between 1KJ1 and selected proteins

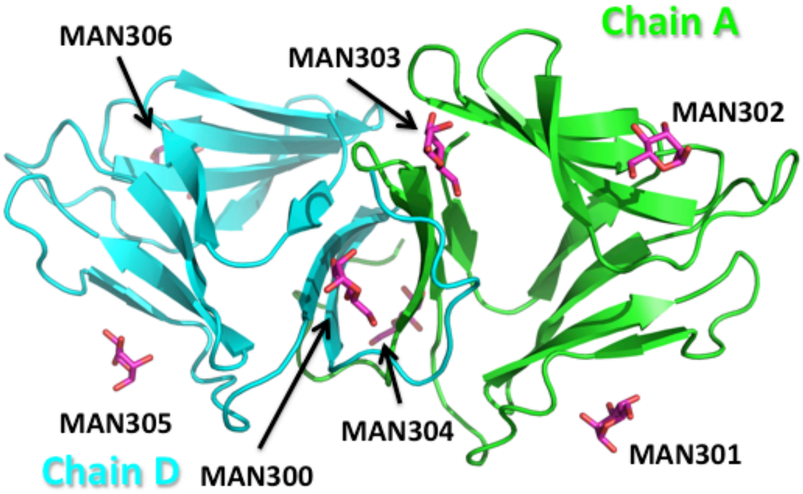

Figure S1. The cartoon structure of garlic lectin dimer protein. The chain A, chain D, and mannoses are colored in green, cyan, and purple, respectively.

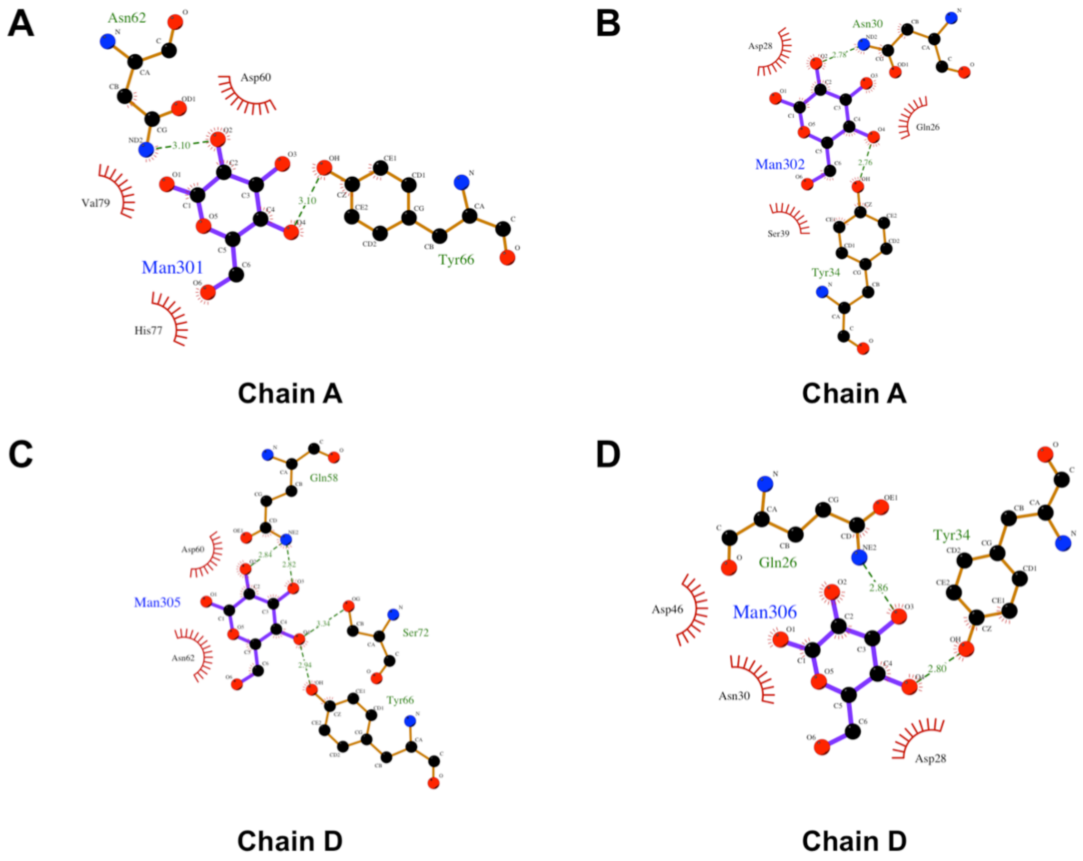

Figure S2. Mannose binding residues. The mannose specific binding residues were identified by using LIGPLOT. The mannose specific binding residues are listed in Table S4.

Single Domain

>1KJ1
RNLLTNGEGLYAGQSLDVEPYHFIMQEDCNLVLYDHSTSVWASNTGILGKKGCKAVLQ
SDGNFVVYDAEGRSLWASHSVRGNGNYVLVLQEDGNVVIYGSDIWSTGTYKRNILMN
DEGLYAGQSLDVEPYHLIMQEDCNLVLYDHSTAVWTTNTDIPGKKGCKAVLQSDGNFV
VYDAEGRSLWASHSVRGNGNYVLVLQEDGNVVIYGSDIWSTNTYK
>UniRef90_A2SVT1
NVLYSPEKLNPGDSLKNGGYSLVMHRGCNLVLYDNSEAIWSSNTNGQGTNCYCTMQR
DGNFVLYSSQGSAIWSSKTWQGGSSHYVLVLQNDRNLVIYGPSRWATETNVLYSPEKL
NPGDSLKNGGYSLVMHRGCNLVLYDNSEAIWSSNTNGQGTNCYCTMQRDGNFVLYSS
QGSAIWSSKTWQGGSSHYVLVLQNDRNLVIYGPSRWATET
>UniRef90_B2KNH9
NHLLPGERLNPGNFLKQDRYMLIMQEDCNLVLYNLNKPEWATKTANQGSRCFVTLQSD
GNFVIYDEHEQEGRNEAIWASKTDGENGNYVIILQKDGNLVLYSKPIFATGTNHLLPGER
LNPGNFLKQDRYMLIMQEDCNLVLYNLNKPEWATKTANQGSRCFVTLQSDGNFVIYDE
HEQEGRNEAIWASKTDGENGNYVIILQKDGNLVLYSKPIFATGT
>UniRef90_B4XS32
RNLLTNGEGLYAGQSLDVEQYKFIMQDDCNLVLYEYSTPIWASNTGVTGKNGCRAVM
QRDGNFVVYDVNGRPVWASNSVRGNGNYILVLQKDRNVVIYESDIWSTGTYRRNLLTN
GEGLYAGQSLDVEQYKFIMQDDCNLVLYEYSTPIWASNTGVTGKNGCRAVMQRDGNF
VVYDVNGRPVWASNSVRGNGNYILVLQKDRNVVIYESDIWSTGTYR
>UniRef90_B7U6V0
NVLYSGDTLSSGQSLTQGSYSLTMQSDCNLVLYDYGRAVWSSGTYNRGYNCVLRMQN
DGNLVIYSNNNAIWASNTGGQQGYFVLILQRDRDVVIYGCPSWATGTNVLYSGDTLSSG
QSLTQGSYSLTMQSDCNLVLYDYGRAVWSSGTYNRGYNCVLRMQNDGNLVIYSNNNA
IWASNTGGQQGYFVLILQRDRDVVIYGCPSWATGT
>UniRef90_C9W8B3
NILYSGETLSPGEFLNYGRYIFIMQEDCNLVLYDVDKPIWATNTGGLARDCHLNMQSDG
NLVVYSQTNDPIWASNTGGENGNYVCVLQKDRNVVIYGTARWATGTYTNILYSGETLS
PGEFLNYGRYIFIMQEDCNLVLYDVDKPIWATNTGGLARDCHLNMQSDGNLVVYSQTN
DPIWASNTGGENGNYVCVLQKDRNVVIYGTARWATGTYT
>UniRef90_E2ICK0
RNVLFSGERLFAGDSLVLGNYVLTMQGDCNLVFYAPAAIWSSGTFNRGYNCYATLLNN
GYFVVYDGAGTPLWWAGRNTGTGYYILILLENRNIVLYGPARWVSGTYRNVLFSGERL
FAGDSLVLGNYVLTMQGDCNLVFYAPAAIWSSGTFNRGYNCYATLLNNGYFVVYDGA
GTPLWWAGRNTGTGYYILILLENRNIVLYGPARWVSGTY
>UniRef90_E7CG22
RNILLNGEGLYAGQSLVIEQYTFIMQQDCNLVLYEYSTPIWASNTGITGKNGCRAVMQA
DGNFVVYDFKGRAVWASNSRRGNGNYILVLQEDRNVVIYGSDIWSTGTYRRNILLNGE
GLYAGQSLVIEQYTFIMQQDCNLVLYEYSTPIWASNTGITGKNGCRAVMQADGNFVVY
DFKGRAVWASNSRRGNGNYILVLQEDRNVVIYGSDIWSTGTYR
>UniRef90_F8SK84
NHLLSGEQLNTGQSLTEGTSQFIIQDDCNLVLYKMNIVKWQSNTTGEGSGCYVTFNKQG
NLVIHNNKNRVIWSSNSSRKQGNYILILQKDGNLVIYSKPIWATGTNHLLSGEQLNTGQS

LTEGTSQFIIQDDCNLVLYKMNIVKWQSNTTGEGSGCYVTFNKQGNLVIHNNKNRVIWS
SNSSRKQGNYILILQKDGNLVIYSKPIWATGT
>UniRef90_F8SVT9
RTTLSSGESLNAPHSLHTGNYVFTMQNDCNLVLYDRDRAVWSTRTHGQGQQCRATMQ
SDGNFVVYDNRNRALWASGTHRPTSGFYILILQADRNVVIYHAGRATWATGTRTTLSSG
ESLNAPHSLHTGNYVFTMQNDCNLVLYDRDRAVWSTRTHGQGQQCRATMQSDGNFV
VYDNRNRALWASGTHRPTSGFYILILQADRNVVIYHAGRATWATGT
>UniRef90_F8UZX3
SGESLQTNQYLESGPYRFIMQGDCNLVLYVNKTRALWSSRTNGQGTACNATLQNNGNL
VVLSGSNVVWTSNSARGPNNYRLIVQSDGNVVIYGAALWATNTSGESLQTNQYLESGP
YRFIMQGDCNLVLYVNKTRALWSSRTNGQGTACNATLQNNGNLVVLSGSNVVWTSNS
ARGPNNYRLIVQSDGNVVIYGAALWATNT
>UniRef90_O24274
NSLTSPNSLPSGHSLNTGSYRLIMQADCNLVVYDSGKPVWATNTGGLARDCRLTMHNN
GNLVIYDRSNSVIWQTNTNEKEDHYVLVLQQDRNVVIYGPAVWATGSNSLTSPNSLPSG
HSLNTGSYRLIMQADCNLVVYDSGKPVWATNTGGLARDCRLTMHNNGNLVIYDRSNS
VIWQTNTNEKEDHYVLVLQQDRNVVIYGPAVWATGS
>UniRef90_O24427
RNILLNGEGLYAGQSLEEGSYKLIMQDDCNLVLFEYSTQVWASNTGVSGRNGCRAVMQ
ADGNFVVYDSNSRAVWASQSRRGNGNYILALQEDRNVVIYGTDIWSTGTYRRNILLNG
EGLYAGQSLEEGSYKLIMQDDCNLVLFEYSTQVWASNTGVSGRNGCRAVMQADGNFV
VYDSNSRAVWASQSRRGNGNYILALQEDRNVVIYGTDIWSTGTYR
>UniRef90_P30617
NILYSGETLSTGEFLNYGSFVFIMQEDCNLVLYDVDKPIWATNTGGLSRSCFLSMQTDG
NLVVYNPSNKPIWASNTGGQNGNYVCILQKDRNVVIYGTDRWATGTNILYSGETLSTGE
FLNYGSFVFIMQEDCNLVLYDVDKPIWATNTGGLSRSCFLSMQTDGNLVVYNPSNKPIW
ASNTGGQNGNYVCILQKDRNVVIYGTDRWATGT
>UniRef90_P49329
NILYSSEVLHENQYISYGPYEFIMQHDCNLVLYESGNPTWASNTGGLALHCRATLQTDG
NLVVQNSANRIIWQSNTGTGTNGDYLLVLQKNGNVVIVGPPIWATGTNILYSSEVLHEN
QYISYGPYEFIMQHDCNLVLYESGNPTWASNTGGLALHCRATLQTDGNLVVQNSANRII
WQSNTGTGTNGDYLLVLQKNGNVVIVGPPIWATGT
>UniRef90_Q0R369
RNILLNGEGLYAGQSLEEGPYRLAMQDDCNLVLYDEYSRPVWASNTGVTGRNGCRAV
MQADGNFVVYDSNSRAVWASNSRKGNGNYILVLQKDRNAVIYGSDIWSTGTYRRNILL
NGEGLYAGQSLEEGPYRLAMQDDCNLVLYDEYSRPVWASNTGVTGRNGCRAVMQAD
GNFVVYDSNSRAVWASNSRKGNGNYILVLQKDRNAVIYGSDIWSTGTYR
>UniRef90_Q38724
RNILGNEQALYADQSLDEGPYKLIMQKDCNLVLFEHNTVRWATKTGSLGHYECYALM
QSDGNFVVHNRNGAIVWASNSRRGNGEYVLVLRENGNVVIYGSDIWSTGTRNILGNEQ
ALYADQSLDEGPYKLIMQKDCNLVLFEHNTVRWATKTGSLGHYECYALMQSDGNFVV
HNRNGAIVWASNSRRGNGEYVLVLRENGNVVIYGSDIWSTGT
>UniRef90_Q38725
RNLLGNGEGLYAGQSLEEGPYKLIMQEDCNLVLYEHSTPVWATNTGVTGRNGCKAVM
QADGNFVVYDSNGRAVWASNSIKGNGNYILVLQNDRNVVIYGSDIWSTGTYRNLLGNG

EGLYAGQSLEEGPYKLIMQEDCNLVLYEHSTPVWATNTGVTGRNGCKAVMQADGNFV
VYDSNGRAVWASNSIKGNGNYILVLQNDRNVVIYGSDIWSTGTY
>UniRef90_Q38783
RNILMNDEGLYAGQSLDVEPYHLIMQEDCNLVLYDHSTAVWTSNTDIPGKKGCKAVLQ
SDGNFVVYDAEGRSLWASHSVRGNGNYVLVLQEDGNVVIYGSDIWSTNTYKRNILMN
DEGLYAGQSLDVEPYHLIMQEDCNLVLYDHSTAVWTSNTDIPGKKGCKAVLQSDGNFV
VYDAEGRSLWASHSVRGNGNYVLVLQEDGNVVIYGSDIWSTNTYK
>UniRef90_Q39512
NHLLSGERLNPGQSLTSGNVDLAMQYDCNLVLYDNGKSIWSSGTYGSGSGCYVTLQTD
GNLVIYDNTNNPLWASNTSGENGNYILILQKDRNLVIYSHPIWATGTNHLLSGERLNPGQ
SLTSGNVDLAMQYDCNLVLYDNGKSIWSSGTYGSGSGCYVTLQTDGNLVIYDNTNNPL
WASNTSGENGNYILILQKDRNLVIYSHPIWATGT
>UniRef90_Q39542
NILYSGETLSPGESLTYGRYDFIIQEDCNLVLYDVDKPIWATNTGGVSSGCHLSMQTDGN
LVVYSPKNNPIWASNTEGENGNYVCVLQKDRNVVIYGTASWATGTNILYSGETLSPGES
LTYGRYDFIIQEDCNLVLYDVDKPIWATNTGGVSSGCHLSMQTDGNLVVYSPKNNPIW
ASNTEGENGNYVCVLQKDRNVVIYGTASWATGT
>UniRef90_Q39544
NILYSGETLSPGESLSHGRYVFIMQEDCNLVLYDVDRPIWATNTGGISHGCHLSMQADG
NLVVYSQRNNPIWASNTGGENDANYVLILQKDRNVVIYGPARWATGTYTNILYSGETLS
PGESLSHGRYVFIMQEDCNLVLYDVDRPIWATNTGGISHGCHLSMQADGNLVVYSQRN
NPIWASNTGGENDANYVLILQKDRNVVIYGPARWATGTYT
>UniRef90_Q39728
NHLLTGQRLTTGSFLTEGGFTFIMQSDCNLVLYDLNRPIWASGTYGKGTGCFLSMQSDG
NLVVYDVRNIAIWASNTARNNGNYLLILQKDRNVVIYSQPIWATTTNHLLTGQRLTTGS
FLTEGGFTFIMQSDCNLVLYDLNRPIWASGTYGKGTGCFLSMQSDGNLVVYDVRNIAIW
ASNTARNNGNYLLILQKDRNVVIYSQPIWATTT
>UniRef90_Q39729
LNAGQSLGTGGSLALGGYIFKIQADCNLVLYDNNRAVWASGTNGRATDCILSMQRDGN
LVIYSGSRVIWASNTNRQSGNYYLILQRDRNVVIYDNSNNAIWATGTLNAGQSLGTGGS
LALGGYIFKIQADCNLVLYDNNRAVWASGTNGRATDCILSMQRDGNLVIYSGSRVIWA
SNTNRQSGNYYLILQRDRNVVIYDNSNNAIWATGT
>UniRef90_Q39903
NILYSGETLSAGEFLNQGNYVFIMQEDCNLVLYDVDKPLWETNTGGLSRRCYLNMQTD
GNLVVYNPSNKPIWASNTGGQNGNYVCILQKDGNIAIYGPAIWATGTNILYSGETLSAG
EFLNQGNYVFIMQEDCNLVLYDVDKPLWETNTGGLSRRCYLNMQTDGNLVVYNPSNK
PIWASNTGGQNGNYVCILQKDGNIAIYGPAIWATGT
>UniRef90_Q39904
ENVLYSGETLPTGGFLSSGSFVFIMQEDCNLVLYNVDKPIWATNTGGLSSDCTLSMQTD
GNLVVYTPSNKPIWASNTDSQNGHYVCILQKDRNVVIYGTDRWATGTYTENVLYSGET
LPTGGFLSSGSFVFIMQEDCNLVLYNVDKPIWATNTGGLSSDCTLSMQTDGNLVVYTPS
NKPIWASNTDSQNGHYVCILQKDRNVVIYGTDRWATGTYT
>UniRef90_Q40231
NTLGGGERLNSGESLTEGACVFIMQEDCNLVLYESSRPTWASGSYHQGSGCYVTLQND
GNLVVYNNRNRAVWASDTVRENIGNVILILQKDHNVVLYSLPIWATGTNTLGGGERLN

SGESLTEGACVFIMQEDCNLVLYESSRPTWASGSYHQGSGCYVTLQNDGNLVVYNNRN
RAVWASDTVRENIGNVILILQKDHNVVLYSLPIWATGT
>UniRef90_Q40233
NHLLGGERLNTGQSLTDGGNAFIMQEDCNLVLYESSRPTWASGTYHRGSGCYLAMQN
DGNLVVYDNRNRAIWASQTDRQNVGTVILILQKDHNVVLYTNPIWATGTNHLLGGERL
NTGQSLTDGGNAFIMQEDCNLVLYESSRPTWASGTYHRGSGCYLAMQNDGNLVVYDN
RNRAIWASQTDRQNVGTVILILQKDHNVVLYTNPIWATGT
>UniRef90_Q40417
NILYSGDTLSTGQFLSYGSYVFIMQEDCNLVLYDVDKPIWATNTGGLSSDCHLSMQTDG
NLVVYSPQNKAIWASNTDGENGHFVCILQKDRNVVIYGTDRWATGTYTNILYSGDTLST
GQFLSYGSYVFIMQEDCNLVLYDVDKPIWATNTGGLSSDCHLSMQTDGNLVVYSPQNK
AIWASNTDGENGHFVCILQKDRNVVIYGTDRWATGTYT
>UniRef90_Q41625
NVLYTGESLYGGQSLTWESYTFIMQTDCNLVLYEGNGSIWASGSNGLGSGCYVTMQKD
GNLVIYSKIGNSVWASQTHQAEGNYVLVLQKDRNVVIYGSSLWATNTNVLYTGESLYG
GQSLTWESYTFIMQTDCNLVLYEGNGSIWASGSNGLGSGCYVTMQKDGNLVIYSKIGNS
VWASQTHQAEGNYVLVLQKDRNVVIYGSSLWATNT
>UniRef90_Q4W660
NILHSGETLSPGEFLSYRSYXFIMQEDCNLVLYBIDKPIWATNTGGLSSGCHLSMQSDGN
LVVYSPQNRPIWASDTGGQNDANYVLILQKDRNVVIYGPARWATGTYTNILHSGETLSP
GEFLSYRSYXFIMQEDCNLVLYBIDKPIWATNTGGLSSGCHLSMQSDGNLVVYSPQNRPI
WASDTGGQNDANYVLILQKDRNVVIYGPARWATGTYT
>UniRef90_Q56V23
KSVLKSGESLAAGESLQYAQYILVMQGDCNLVLYANKVKVLWASRTNGKGGAASCKL
SMQNDGNLVIYAATTPVWASRTSRAFASYKLNLQGDGNVVIYGPSGAIWATNTKSVLK
SGESLAAGESLQYAQYILVMQGDCNLVLYANKVKVLWASRTNGKGGAASCKLSMQN
DGNLVIYAATTPVWASRTSRAFASYKLNLQGDGNVVIYGPSGAIWATNT
>UniRef90_Q5EER7
NSLTSPNSLPSGHSLNSDSYRLIMQGDCNFVLYDSGSPVWASNTGGLGSDCRLTLHNNG
NLVIYDRGNNVIWETKTNGKDDHYVLVLQQDRNVVIYGPAVWATGSNSLTSPNSLPSG
HSLNSDSYRLIMQGDCNFVLYDSGSPVWASNTGGLGSDCRLTLHNNGNLVIYDRGNNV
IWETKTNGKDDHYVLVLQQDRNVVIYGPAVWATGS
>UniRef90_Q5MK12
ENILYSGETLSPGDFLSYGSYVFIMQEDCNLVLYNVDKPIWATNTGGLSRDCHLSMQSD
GNLVVYSRQNKPIWASDTGGENGNYVCVLQKDRNVVIYGTARWATGTENILYSGETLS
PGDFLSYGSYVFIMQEDCNLVLYNVDKPIWATNTGGLSRDCHLSMQSDGNLVVYSRQN
KPIWASDTGGENGNYVCVLQKDRNVVIYGTARWATGT
>UniRef90_Q5W956
ILYSGDSLRSGQSLTYASYNFIMQNDCNLVQYDNGKAIWASGTNGRGSGCYCAMQSDG
NLVVYTSSNKAVWASNTNVGNGHYVLILQKDRNVVIYGGARWATNTILYSGDSLRSGQ
SLTYASYNFIMQNDCNLVQYDNGKAIWASGTNGRGSGCYCAMQSDGNLVVYTSSNKA
VWASNTNVGNGHYVLILQKDRNVVIYGGARWATNT
>UniRef90_Q679P2
RNVLVNNEGLYAGQSLVEEQYTFYMQDDCNLVLYEYSTPIWASNTGVTGKNGCRAVM
QADGNFVVYDVKGRAVWASNSRRGNGNYILVLQKDRNVVIYGSDIWSTGTYRRNVLV

NNEGLYAGQSLVEEQYTFYMQDDCNLVLYEYSTPIWASNTGVTGKNGCRAVMQADGN
FVVYDVKGRAVWASNSRRGNGNYILVLQKDRNVVIYGSDIWSTGTYR
>UniRef90_Q6JTD2
EDIMFNGESLSTNEFLENGPYRFIMQDDCNLVLYINRTRALWASGTNGRGSNCRATLQN
NGNLVVITGSDIVWSSNSSRGQNSYRLIVQSDGNVVIYGGALWATNTEDIMFNGESLST
NEFLENGPYRFIMQDDCNLVLYINRTRALWASGTNGRGSNCRATLQNNGNLVVITGSDI
VWSSNSSRGQNSYRLIVQSDGNVVIYGGALWATNT
>UniRef90_Q6SQE5
NVLYSGQALSTGASLSFAAYQFVMQADCNLVLYDNGNPVWASGTNGRGSGCQCRMQ
YDGNLVVYTSGNRAVWASNTNRGTGRYQCILQRDRNVVVYDPQGRPIWATGTNVLYS
GQALSTGASLSFAAYQFVMQADCNLVLYDNGNPVWASGTNGRGSGCQCRMQYDGNL
VVYTSGNRAVWASNTNRGTGRYQCILQRDRNVVVYDPQGRPIWATGT
>UniRef90_Q6V8L5
LNSNASLNTGDYLSVKAYKFIMQEDCNLVLYDAGTAIWSSGTYGRGYGCRCTMQDDG
NLVVYNSRSEAVWSSRTYRAKGFYILVLQGDRNVVIYSRGAAIWSTGTYLNSNASLNT
GDYLSVKAYKFIMQEDCNLVLYDAGTAIWSSGTYGRGYGCRCTMQDDGNLVVYNSRS
EAVWSSRTYRAKGFYILVLQGDRNVVIYSRGAAIWSTGTY
>UniRef90_Q6XCI6
NVLQSGQQLGTGGSLDYAAYNFRMQADCNLVLYDNGNAVWASGTNGRGSGCTCRMQ
YDGNLVVYTNANRAVWASNTNRGTGSYQLILQRDRNVVIYDPSGRAIWATGTNVLQS
GQQLGTGGSLDYAAYNFRMQADCNLVLYDNGNAVWASGTNGRGSGCTCRMQYDGN
LVVYTNANRAVWASNTNRGTGSYQLILQRDRNVVIYDPSGRAIWATGT
>UniRef90_Q7Y041
NILYSGETLSAGESLNYGPYTFIMQQDCNLVLYDVDKPIWASNTGGLARGCHLSMQSD
GNLVVYSPSGNRPIWASNTQGENGNYVCIVQKDRNVVIYGTARWATGTYTNILYSGET
LSAGESLNYGPYTFIMQQDCNLVLYDVDKPIWASNTGGLARGCHLSMQSDGNLVVYSP
SGNRPIWASNTQGENGNYVCIVQKDRNVVIYGTARWATGTYT
>UniRef90_Q7Y1W7
NILYSGETLSTGESFNYGPYTFIMQEDCNLVLYDVDKPIWASNTGGLARGCHLSMQSDG
NLVVYTPSGNRAIWASNTQGENGNYVCILQKDRNVVIYGTARWATGTNILYSGETLSTG
ESFNYGPYTFIMQEDCNLVLYDVDKPIWASNTGGLARGCHLSMQSDGNLVVYTPSGNR
AIWASNTQGENGNYVCILQKDRNVVIYGTARWATGT
>UniRef90_Q8H2A4
ENILYTNDVLFGGQSLTNGNFRFTMQTDCNLVLYDGGSAIWSSGTYRKGTGCYVKMQS
DGNLVVYNGSNNALWASNTDRGRGNYVCILQRDRNVVVYGPALWATGTENILYTNDV
LFGGQSLTNGNFRFTMQTDCNLVLYDGGSAIWSSGTYRKGTGCYVKMQSDGNLVVYN
GSNNALWASNTDRGRGNYVCILQRDRNVVVYGPALWATGT
>UniRef90_Q8L568
NSLSSPNSLFTGHSLEVGPSYRLIMPGDCNFVLYDSGKPVWASNTGGLGSGCRLTLHNN
GNLVIYDQSNRVIWQTKTNGKEDHYVLVLQQDRNVVIYGPVVWATGSNSLSSPNSLFT
GHSLEVGPSYRLIMPGDCNFVLYDSGKPVWASNTGGLGSGCRLTLHNNGNLVIYDQSN
RVIWQTKTNGKEDHYVLVLQQDRNVVIYGPVVWATGS
>UniRef90_Q8LK14
ILYSGETLSAGQSLNNGPYTFIMQEDCNLVLYNVDKPIWASNTGGLARGCHLSMQRDG
NLVVYSQRNRPIWASNTGGHNAANYVLILQKDRNVVIYGPAKWATGTYTILYSGETLS

AGQSLNNGPYTFIMQEDCNLVLYNVDKPIWASNTGGLARGCHLSMQRDGNLVVYSQR
NRPIWASNTGGHNAANYVLILQKDRNVVIYGPAKWATGTYT
>UniRef90_Q945J7
ENILYSGETLPTGGFLTSGSFVFIMQQDCNLVLYNIDKPIWATNTGGLSSDCSLSMQTDG
NLVVYNPSNKPIWASNTDGQNGNYVCILQKDRNVVIYGTNRWATGTENILYSGETLPTG
GFLTSGSFVFIMQQDCNLVLYNIDKPIWATNTGGLSSDCSLSMQTDGNLVVYNPSNKPI
WASNTDGQNGNYVCILQKDRNVVIYGTNRWATGT

Double Domain

>1KJ1
RNLLTNGEGLYAGQSLDVEPYHFIMQEDCNLVLYDHSTSVWASNTGILGKKGCKAVLQ
SDGNFVVYDAEGRSLWASHSVRGNGNYVLVLQEDGNVVIYGSDIWSTGTYKRNILMN
DEGLYAGQSLDVEPYHLIMQEDCNLVLYDHSTAVWTTNTDIPGKKGCKAVLQSDGNFV
VYDAEGRSLWASHSVRGNGNYVLVLQEDGNVVIYGSDIWSTNTYK
>UniRef90_A2IBI1
EQVLIANRTLYAGQSLYSMNRNYRFIMQWDCSLALYEFQKPLWISTTDRQPGTFCDATL
DAGSARLAVTQRNTNKQVWSSTTSFYAFKYVLVLQTDLNVVIYSRPVWSTNTFRFIMQ
DDCDLVLYDDDRRIWSSETSGKGTGCNAKLNGLTGQLIVRDNNGRIVWSSKNRAVPIK
KHILLLQPDRNVVVYTGRVWASDT
>UniRef90_A2ICN3
NYLLTGETLNTNEHLRNGDFDLVMQEDCNLVLYNGNWQSNTANRGKECKLTLTDRGE
LLIQDRDGSNVWSSGSQSERGNYAAVLHPEGRLVIYGNMLFSGQILHNDGMLTARNHR
LVMQEDCNLVLYGGKLGWQSNTHGNGEHCFLRLTHKGELIIKDDDFKTIWSSRSKSKQ
GDYAFILQDDGFAVIYGPAIFSTGS
>UniRef90_B1A014
NYLLSGQTLDTDGHLKNGDFDFVMQDDCNAVLYNGNWQSNTANRGRDCKLTLTDNG
ELIINDGKGSTVWKSGSPSVRGNYAAAVHPEGRLVIYGPSVFNNMLFSGQVLHEDGRLT
VKNHRLVMQGDCNLVLYGGKFGWQSNTHGNGEHCFVRLNHKGELIIKDGDFKTIWSS
RTSSKQGEYVFILQDNGFGVVYGPAIWAT
>UniRef90_B5LYJ9
LLSGQTLDTEGHLKNGDFDLVMQDDCNLVLYNGNWQSNTANNGRDCKLTLTDYGELV
IKNGDGSTVWKSGAQSVKGNYAAVVHPDGRLVVLGNLLFSGQVLYGDGRLTAKNHQL
VMQGDCNLVLYGGKYGWQSNTHGNGEHCFLRLNHKGELIIKDDDFKTIWSSRSSSKQG
EYVLILQDDGFGVIYGPAIFET
>UniRef90_D4P965
NYLLSGQTLDTDGHLKNGDFDFVMQDDCNAVLYNGGWQSNTANKGRDCKLTLTDRG
ELVINNGGGSTVWRSGSQSVKGNYAAVLHPEGKLVIYGNMLFSGQVLYGDGKITARNH
MLVMQGDCNLVLYGGKYGWQSNTHGNGEHCFLRLNHKGELIIKDDDFKSIWSSQSSSK
QGDYVFILQDDGFAVIYGPAMWAT
>UniRef90_E2ITD6
NHLLSGETLDTNGHLRNSDFDLIMQEDCNAVLYNGNWQSDTANKGRDCKLTLTDRGE
LVIKNGDRSIVFRSGSQSDVRGNYALVVHPEGRLVIYGNMLFSGQVLYGDGKLRARNH
MLVMQGDCNLVLYGGKYGWQSNTHGNGENCFVRLSHKGELIIKDDDFQTIWSSRSGSK
QGDYVFILQEDGFAVIYGPAIWAT
>UniRef90_H1A7C5
NYLLSGETLGTDQQLNYDKLSFVMQGDCNLVLYNGGWQSNTANRGKDCTVSLNNDG
QLVIKSGDGKTVWTSGDSSPRGNYAAVLHPEGKVVVYGPEVWSANLMFSGQVLYRDG
KLTTRDYMFVMQGDCNLVLYTHGGWQSNTHGNGQHCFLRLDHRGMLTIKDDSYGTV
WTSGQKSAEGEYVLILQEDAKVVVYGPAIWST
>UniRef90_H8Y197
NHLLSGEILDTNGHLRNGDFDLVMQEDCNAVLYNGNWQSNTANKGRDCKLTLTNRGE
LIIKNGDGSIVFRSGSQSERGDYALVVHPEGKLVIYGNMLFSGQVLHEDGRLTARNHKL

VMQGDCNLVLYGGKFGWQSNTHGNGDHCFLRLSHKGELIIKDDNFKTIWSSQSSSKQG
DYVFILQENGFGVIYGPAIFET
>UniRef90_P86626
DNNVLLTGDVIHTDNQLSYESAAFVMQGDCNLVLYNEAGGFQSNTHGRGVDCTLRLN
NRGQLEIHSANSNTPVWVYPRSVNTVRGNYAATLGPDQHVTIYGPAIWSTPAAARNVL
FSSQVMYDNAQLATRDYSLVMRDDCNLVLTKGSKTNIVWESGTSGRGQHCFMRLGHS
GELDITDDRLNTVFVSNTVGQEGDYVLILQINGQAVVYGPAVWST
>UniRef90_P92932
RNVLTTGETLHAGEHLDIGQYTLSMRYDCNLVLYENSNPLWASNTQYEENVVCQVVLR
KDGILVVSYVGSEADYPVWWSSNDRLDSGNYALVLHEDGNLVIYGTDIWSTGTAKRSV
LKSGEQLSSEQFLNTQQFKLIAQEDCNLVLYEYNIVIWASNTVVGAHAGCRAAMQVDG
NFVIYFNLHAIWSSHTDRENGNYVLVLQQDGNVVIYGPDVWSTGT
>UniRef90_Q2LAE3
NYLLSGETLDTNGHLRNGNFDLVMQEDCNAVLYNGGWQSNTANRGRDCKLSLTDYGE
LVIKNGDGSTVWRSGSQSDKGKYAAVVHPDGRLVVYGPSVFNNMLFSEQVLYEDGRL
TAKNHRLVMQGDCNLVLYGGKFGWQSNTHGNGEDCFVRLNHKGELVIKHDNFRTIWS
SQQNSNEGDYVFILQDDGFGVIYGPAIWAT
>UniRef90_Q38731
NYMLSGETLNTDGRLTNGELTLIMQVDCNLVLYHGGWQSNTANNGRDCKLTLTDFGE
LVIKSGTGSTVWSSGSKQSMKGNYAAVVHPEGRLVIYGNMLFSGQVLYGDGTLSARNH
KLVMQGDCNMVLYGGKYGWQSNTHGNGKYCFAMLTHKGELVIRDDDFKSVWSSKSS
SKQGDYVFILQDDSLAIIYGPAVFSTGS
>UniRef90_Q38787
RNLLTNGEGLYAGQSLNVEPYHFIMQEDCNLVLYDHSTAVWATNTDIPGKKECRAVLQ
SDGNFVVYDADGRPLWASHSVRGNGNYVLVLQEDGNVVIYGSDIWSTGTYRNILRNDE
GLYAGQSLDVEPYHFIMHEDCNLVLYDHSTAVWASNTDIPGKKGCKAVLQSDGNFVV
YDAEGRSLWASHSVRGNGNYVLVLQEDGNVVIYGSDIWSTDTYR
>UniRef90_Q6Q5X6
NYLLSGETLNTNGHLRNGDFDLVMQEDCNAVLYNGKWQSNTANKGRDCKLTLTNRGE
LVIKNGDGSTVFSSGSQSDMRGNYALVVHPDGRLVIYGNMLFSGQVLYSDGMLVARN
HRLVMQGDCNLVLYGGKFGWQSNTHGNGEHCFVRLNHKGELVIKDDNFQTIWSSRSSS
AKQGDYVFILQDDGFGVTYGPAIWET
>UniRef90_Q9FVA1
NVLLTGDVLHTDNQLSFESAAFVMQGDCNLVLYNEAGGFQSNTHGRGVGCTLRLNNF
GQLEIHSANSNTPVWVSLRNGIPVRGDYAAVLGPDQHVTIYGPAIWSTRNVLFSSQVMS
DNAQLATRDYSLVMRDDCNLALTKGSKTNIVWESGTSGRGQHCFMRLGHTGLIEISDD
RLNTVWRSNTVGQEGDYVLILQINGQAVVYGPAVWST
